# Are cases of enteric disease due to *Salmonella, Campylobacter, Giardia*, and *Cryptosporidium* associated with well water?

**DOI:** 10.1097/EE9.0000000000000499

**Published:** 2026-06-16

**Authors:** Miriam Wamsley, Kevin Henry, Robin Taylor Wilson, Eric Coker, Heather M. Murphy

**Affiliations:** a Department of Epidemiology and Biostatistics, Temple University, Philadelphia, Pennsylvania; b Department of Geography, Environment, and Urban Studies, Temple University, Philadelphia, Pennsylvania; c British Columbia Centre for Disease Control, Vancouver, British Columbia, Canada; d Water, Health and Applied Microbiology Lab, Department of Pathobiology, University of Guelph, Guelph, Ontario, Canada.

**Keywords:** *Cryptosporidium*, *Giardia*, *Salmonella*, *Campylobacter*, groundwater, enteric disease, karst, private well, spatial, temporal

## Abstract

**Background:**

*Campylobacter*, non-typhoidal *Salmonella*, *Shigella*, *Cryptosporidium*, and *Giardia* are responsible for ~1 million domestically acquired waterborne illnesses annually in the United States. The contribution of private well water and underlying geology to these infections has been underexplored. The objectives of this research were to (1) determine whether enteric disease cases in Pennsylvania cluster in time and space; and (2) determine whether enteric disease cases are associated with private well water and karst geology.

**Methods:**

Confirmed cases of *Campylobacter*, non-typhoidal *Salmonella*, *Cryptosporidium*, and *Giardia* from 2010 to 2019 in Pennsylvania were analyzed. Spatial clusters were identified using a Poisson-based spatial scan statistic (SaTScan), and temporal patterns were examined using the R package *Model Temporal Trends*. Zero-inflated negative binomial model regression with county-level random intercepts examined associations between disease incidence, private well usage, and karst geology.

**Results:**

All four pathogens had significant clusters of illness in time and space. *Cryptosporidium*, *Giardia*, and *Campylobacter* cases were significantly associated with areas served by private wells (*P* < 0.05; *P* < 0.001), and cases of *Cryptosporidium* and *Campylobacter* were associated with karst geology (*P* < 0.01).

**Conclusions:**

This novel investigation adds to a growing body of evidence that private well water is a risk factor for enteric disease. Public health interventions should target the management of private well water to reduce the burden of disease globally, particularly in communities not serviced by public water supplies.

**What this study adds**

This novel investigation adds to a growing body of epidemiological evidence that private well water was associated with an increased incidence of enteric disease and that geology could be influencing exposure risk and transport of pathogens to groundwater sources. We conducted a robust spatial epidemiological study of four reportable enteric diseases and the relationship between private well ownership and underlying geology. This work is important not only for the 42.5 million Americans who rely on untreated, unregulated private well water for consumption but also for the estimated 3 billion people worldwide who use groundwater for their daily needs.

## Background

According to the Centers for Disease Control and Prevention (CDC)’s most recent estimate of infectious waterborne disease burden, the US population experiences 7.15 million waterborne illnesses, which result in $3.33 billion in direct health care costs annually.^1^ *Campylobacter*, nontyphoidal *Salmonella*, *Shigella*, *Cryptosporidium*, and *Giardia* collectively are estimated to be responsible for about 1 million annually acquired waterborne illnesses in the United States, after adjusting for estimated underreporting, underdiagnosis, and international travel.^1^ Half of US waterborne disease outbreaks have been linked to drinking untreated or undertreated groundwater.^2^ The state of Pennsylvania (PA) has the second highest number of acute gastrointestinal (AGI) outbreaks linked to groundwater each year.^3^ Using estimates from Murphy et al.,^4^ we estimate that 1.27 million people in the United States experience AGI each year due to untreated and unregulated private well water.

Approximately 3.5 million people (25% of people) use a private well for drinking water in PA.^5^ This proportion is higher than the US national average of 13% of homes (~42.5 million people) relying on a private well.^6^ In the United States, private wells are unregulated and are defined as systems serving fewer than 25 individuals.^7^ Private well owners in PA and across the United States are responsible for the protection, maintenance, testing, and treatment of their well water.

There is a common misconception that well water is free of microbial contamination because it originates from the subsurface; however, enteric pathogens have been found in well water supplies across the United States and other countries around the world.^8^ For example, a recent study in Wisconsin found pathogens in 48% of the wells that they sampled (n = 138),^9^ and in a Minnesota study,^10^ *Cryptosporidium* was found in 40% of 145 wells sampled. Key causes of well water contamination include agricultural land use and livestock, inadequate well construction, rainfall, underlying geology, septic systems, and leaking wastewater systems.^3,11^

Few studies have examined the spatiotemporal links between enteric disease and private well use. Prior research in Maryland, Massachusetts, and British Columbia (BC), Canada, has reported higher disease incidence among populations relying on private wells. Murray et al. assessed the relationship between *Campylobacter* and private wells in Maryland and stated that spatial regression analysis should be employed to assess this relationship further.^12,13^ A study in BC, Canada, focused on the spatial distribution of campylobacteriosis, cryptosporidiosis, giardiasis, salmonellosis, and verotoxigenic *Escherichia coli* found that disease risk was 5.2 times higher for those living on land parcels served by private wells than the municipal water system.^14^ A more recent study, also in BC, Canada, found similar results: that those served by private well water had higher odds of contracting campylobacteriosis than those served by municipal water supplies.^15^ In Massachusetts, United States, rates of giardiasis and cryptosporidiosis were highest for people served by a mixed unfiltered surface water and groundwater.^16^

Geology has been identified in several reviews as an important risk factor for pathogen presence in well water supplies^3,8,11^; however, few population-based health studies have examined this link explicitly.^11,17^ Aquifers in fractured rock, gravel, and karst systems are particularly vulnerable to contamination, as cracks in these rock formations can facilitate rapid transport of microbial contaminants into groundwater.^18,19^ Karst is a subsurface geology type made up of dissolvable rock formations such as limestone or marble. These formations erode over time and develop macropores or caves, which can hold a significant amount of water. Areas with karst can have sinkholes and springs. Water can move through these macropores or caves much faster than through geologies with smaller pore space.^20^

To fill knowledge gaps around whether well water contributes to enteric disease, the goals of the present study were to (1) use spatial and temporal methods to identify if cases of *Campylobacter*, *Salmonella*, *Cryptosporidium*, and *Giardia* clustered in time and space in PA, and (2) investigate if these clusters of illness were associated with private well use and the underlying geology.

## Methods

### Data sources

#### Case data

Confirmed cases for PA for the years 2010–2019 (salmonellosis n = 9,924; campylobacteriosis n = 15,854; giardiasis n = 4,537; and cryptosporidiosis n = 4,017) were obtained from the Pennsylvania Electronic Disease Reporting System. Case data were geocoded to the zip code tabulation area (ZCTA) level using reported zip code and county information. The specimen collection date served as the primary temporal marker for illness onset; when unavailable, the initial reporting date to the Commonwealth of Pennsylvania was substituted. Cases lacking a county designation or reporting any travel during the exposure period were excluded from the analysis. Philadelphia County data were not available for inclusion in this study. Study approval was obtained from both the Pennsylvania Department of Health and Temple University’s Institutional Review Boards (Protocol #28345).

#### Community data

Population estimates for each ZCTA for each year were collected from the Census Bureau,^21^ using the censusapi package version 0.8.1 for R.^22^ Decadal census data were used for 2010, and the American Community Survey 5-year estimates were analyzed for years 2011–2019. Polygons for the geographic area of the five-digit ZCTA and for counties in PA were obtained from the 2010 US Census.^21^ Polygons (ZCTAs and counties) were matched to populations (population with zip codes) using the zipcodeR package version 0.3.5.^23^

Public water system (PWS) boundaries for PA from 2018 were provided as a shapefile from the PA Department of Environment.^24^ To estimate the proportion of a population using private wells by county, we assumed geographies without publicly supplied water-use private wells. The area of a county using private well water was estimated by removing the area served by public water supplies (the PWS shapefile) from the total area of a county using the county Tiger line shapefile^25^ and the erase tool in ArcGIS Pro version 3.1.

**Figure 1.**
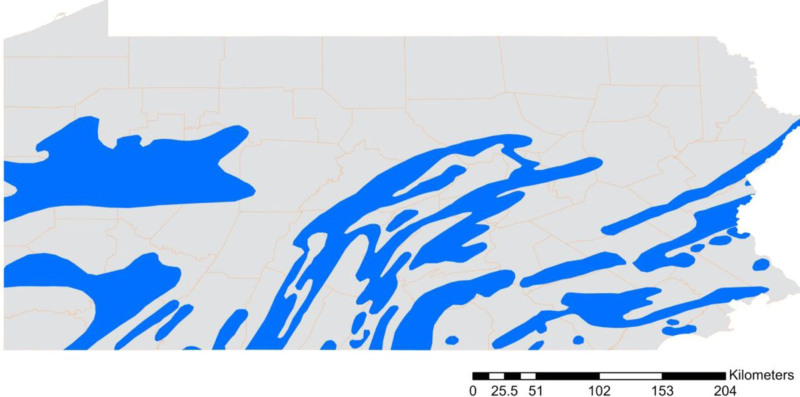
Area of Karst in PA (carbonate) (USGS, 2004).^26^

The area of a county underlain by karst geology was estimated using the US Geological Survey (USGS) karst shapefile^26^ (Figure 1). The proportion of a county underlain by karst was calculated by dividing the area of karst by the total area of the county. All calculations were completed in ArcGIS Pro.^27^ The range of area underlain by karst in PA per county was 0%–76.8% (median: 19.8%; mean: 25.6%).

### Spatial analysis

Incidence rate ratios (IRRs) were calculated at the county level and compared with the statewide average rate for the study period. These relationships were visualized using ArcGIS Pro 3.1. Counties with fewer than six cases were censored in accordance with data use agreements. To examine incidence rate variability at finer geographic resolutions, we applied kriging interpolation (ArcGIS Pro 3.1 spatial analysis tools) to smooth and visualize ZCTA-level rates.

To detect spatial clusters of enteric disease cases in Pennsylvania between 2010 and 2019, we performed a spatial-only analysis using SaTScan software, comparing the observed number of reported illnesses for each pathogen in each ZCTA to the statewide illness rate.^28,29^ Details of the SaTScan methods can be found in Text S1 https://links.lww.com/EE/A436.

### Temporal Analysis

Ten years of weekly case counts were used to assess temporal patterns and seasonality. Complete details are available in Text S2 https://links.lww.com/EE/A436. Briefly, to characterize the association between seasonal variation and illness counts for each pathogen, we employed a zero-inflated negative binomial regression model.

To estimate the occurrence and frequency of outbreaks, an outbreak was defined as a week in which observed case counts exceeded model predictions. A negative binomial regression model was fit using the Model Temporal Trending package (version 0.0.3) with 10 years of weekly count data for each pathogen.^30^ Weekly estimates were plotted with corresponding prediction intervals from the negative binomial model outputs. An outbreak was defined as a week in which illness counts exceeded the upper bound of the 95% prediction interval.^31^

### Regression Analysis

An ecological study design utilizing multiple zero-inflated negative binomial regression models with random intercepts for county was utilized to assess the effect of the percentage of county area underlain by karst geology and the percentage of geographic area lacking access to a public water supply (i.e., reliance on private wells) on weekly counts of new illness cases by county for each pathogen. When both the percentage of area served by private wells and the percentage of area underlain by karst were statistically significant, we assessed their interaction effect on county disease counts. These models were fitted separately for each pathogen. The details of the model are presented in Text S3 https://links.lww.com/EE/A436.

Model fit was evaluated using the Akaike information criterion, with the model exhibiting the lowest Akaike Information Criterion selected as the best-fitting model (Table S1 https://links.lww.com/EE/A436). We calculated IRRs and their corresponding 95% confidence intervals. All analyses and visualizations were performed in R version 4.2.2 (R Core Team, 2022).

## Results

The proportion of an area not served by a public water supply (our proxy for private well reliance) per county in Pennsylvania ranged from 9.3% to 99.7% of households (median: 89.7%; mean: 82.4%) (Table S2 https://links.lww.com/EE/A436). Spatial cluster analysis revealed statistically significant clustering for all pathogens examined.

**Figure 2.**
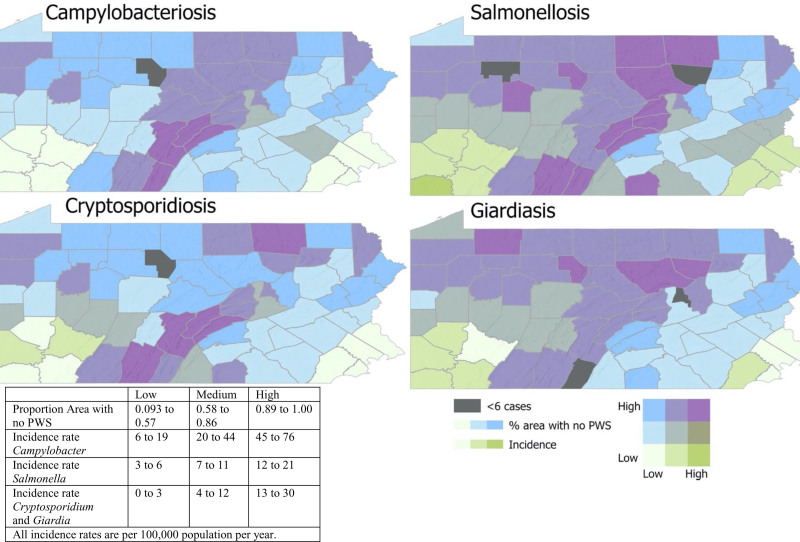
Bivariate color maps. Areas with private wells (not served by a public water supply (PWS) and annual incidence of *Campylobacter*, *Salmonella*, *Cryptosporidium*, and *Giardia* in PA (excluding Philadelphia). (circa 2010–2019) (Purple color indicates a high proportion of area not served by public water and a high incidence of enteric disease, where pale green indicates a low proportion of area served by public water and a low incidence of enteric disease) (results excluding Philadelphia) (excluding Philadelphia) (circa 2010–2019) (Darker blue shading indicates a higher proportion of private well usage, while darker green represents elevated illness rates. Counties exhibiting both high incidence rates and substantial private well coverage appear in dark purple.) (results excluding Philadelphia.)

Bivariate choropleth maps illustrate the relationship between disease incidence and the proportion of county area served by private wells (Figure 2). Spatial clusters demonstrated varying ranges of relative risk: *Salmonella* (1.31–2.70) and *Campylobacter* (1.62–3.31) showed narrower ranges compared with *Giardia* (1.86–10.35) and *Cryptosporidium* (1.93–5.04). The highest population attributable fractions (PAF) were observed for *Cryptosporidium* cluster 2 (PAF = 11.2%), located southwest of Harrisburg, PA, and *Campylobacter* cluster 2 (PAF = 5.3%), located northwest of Harrisburg, PA. Notably, the five highest-ranking *Cryptosporidium* spatial clusters (clusters 2–6) yielded a combined PAF of 24.1% (Table S3 https://links.lww.com/EE/A436). Detailed results of the spatial cluster analysis can be found in the Text S1 https://links.lww.com/EE/A436.

### Spatial Clusters

#### Campylobacter

**Figure 3.**
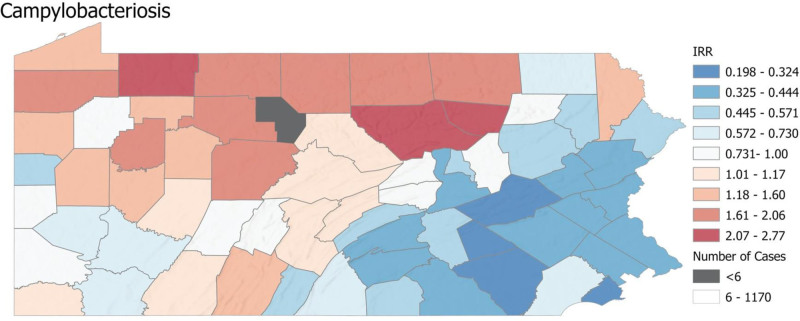
Incidence rate ratios of Campylobacteriosis cases across PA by county (excluding Philadelphia) (circa 2010–2019).

SaTScan analysis identified nine statistically significant spatial clusters exhibiting higher-than-expected campylobacteriosis incidence relative to the statewide average (Figure S1 https://links.lww.com/EE/A436). All clusters demonstrated statistical significance (*P* < 0.001), with relative risks ranging from 1.62 (cluster 9) to 3.31 (cluster 2) (Table S3 https://links.lww.com/EE/A436). Three clusters with radii exceeding 50 km were concentrated in the northern region of the state. These clusters correspond to areas of elevated incidence rates depicted in Figures S1 and S2 https://links.lww.com/EE/A436 and Figure 3.

#### Salmonella

**Figure 4.**
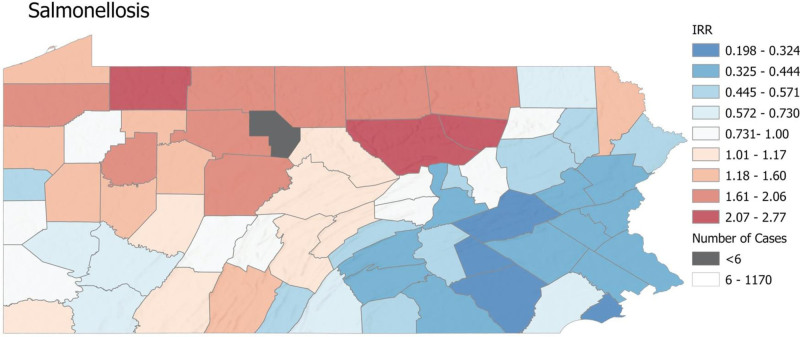
Incidence rate ratios of Salmonellosis cases across PA by county (excluding Philadelphia) (circa 2010–2019).

Six spatial clusters of elevated salmonellosis incidence were identified (Figure S1 https://links.lww.com/EE/A436). One particularly large cluster, with a radius exceeding 100 km, was in the north-central region (Table S3 https://links.lww.com/EE/A436). Kriged surfaces revealed smaller-scale spatial variability throughout the state (Figure S2 https://links.lww.com/EE/A436), with Figure 4 demonstrating lower county-level incidence rates in the southeastern region.

#### Cryptosporidium

**Figure 5.**
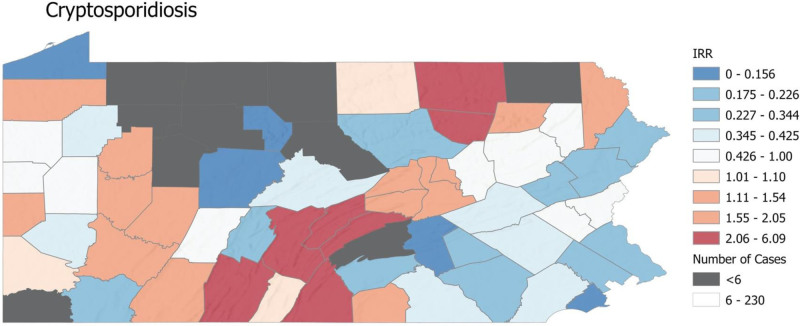
Incidence rate ratios of Cryptosporidiosis cases across PA by county (excluding Philadelphia) (circa 2010–2019).

SaTScan analysis identified ten statistically significant clusters of cryptosporidiosis (*P* < 0.1) (Figure S1 https://links.lww.com/EE/A436). These clusters were concentrated in the western and central portions of the state, including rural areas surrounding Pittsburgh and regions north and west of Harrisburg (Table S3 https://links.lww.com/EE/A436). The spatial distribution of smoothed ZCTA-level incidence rates (Figure S2 https://links.lww.com/EE/A436) corresponded closely with SaTScan cluster locations and county-level IRRs (Figure 5), particularly elevated rates in the south-central region (corresponding to clusters 2 and 6) and the northeastern quadrant (corresponding to cluster 4).

#### Giardia

**Figure 6.**
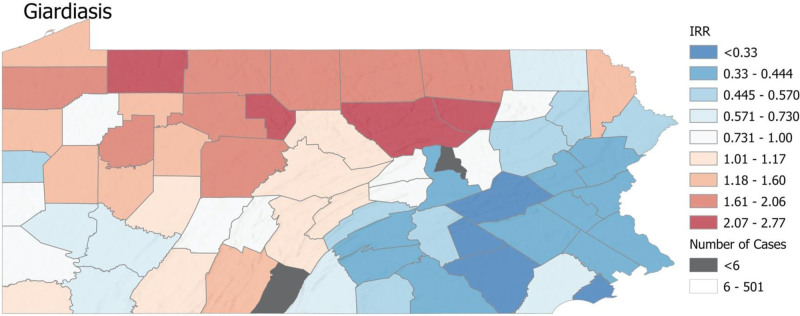
Incidence rate ratio of Giardiasis cases across PA by county (excluding Philadelphia) (circa 2010–2019).

Thirteen statistically significant spatial clusters of giardiasis were identified (*P* < 0.05) (Figure S1 https://links.lww.com/EE/A436, Table S3 https://links.lww.com/EE/A436). Relative risks ranged from 1.86 (cluster 15) to 10.35 (cluster 17). The highest burden of giardiasis in PA was concentrated in the northwestern quadrant (Figure 6 and Figure S2 https://links.lww.com/EE/A436).

### Temporal results

Zero-inflated negative binomial models were employed to assess the association between statewide weekly case counts for all four pathogens (2010–2019) and seasonal variation, with spring (March–May) serving as the reference category. All pathogens exhibited peak incidence rates during summer months (June–August): *Campylobacter* IRR = 1.68 (95% CI: 1.58, 1.81); *Salmonella* IRR = 1.88 (95% CI: 1.75, 2.03); *Cryptosporidium* IRR = 2.03 (95% CI: 1.79, 2.29); and *Giardia* IRR = 1.64 (95% CI: 1.49, 1.82). Winter (December–February) demonstrated the lowest illness rates for all pathogens, except for *Giardia*, which showed no statistically significant difference (*Campylobacter* IRR = 0.81 [95% CI: 0.75, 0.88]; *Salmonella* IRR = 0.59 [95% CI: 0.53, 0.65]; *Cryptosporidium* IRR = 0.87 [95% CI: 0.76, 1.00]) (Table S3 https://links.lww.com/EE/A436). Fall (September–November) incidence rates generally exceeded spring rates, except for *Salmonella (Campylobacter* IRR = 1.12 [95% CI: 1.04, 1.22]; *Cryptosporidium* IRR = 1.20 [95% CI: 1.06, 1.37]; *Giardia* IRR = 1.64 [95% CI: 1.48, 1.81]).

Outbreak detection analysis, defined as weeks with case counts exceeding the 95th percentile prediction interval, identified the following number of outbreaks: 6 for campylobacteriosis, 12 for salmonellosis, 2 for cryptosporidiosis, and 5 for giardiasis (Figures S3–S6 https://links.lww.com/EE/A436). Individual outbreak characteristics and prediction intervals are detailed in Table 1. Peak outbreak weeks across all four pathogens occurred most frequently during weeks 52 and 26 (Table S4 https://links.lww.com/EE/A436).

**Table 1.** Zero-inflated negative binomial model results for the association between weekly cases and Season in PA between 2010 and 2019

Season	*Campylobacter* ^a^		*Salmonella* ^a^		*Cryptosporidium* ^b^		*Giardia* ^b^	
	IRR	95% CI	IRR	95% CI	IRR	95% CI	IRR	95% CI
Spring (MAM)	Ref.	Ref.	Ref.	Ref.	Ref.	Ref.	Ref.	Ref.
Summer (JJA)	**1.68** ***	1.58, 1.81	**1.88** ***	1.75, 2.03	**2.03** ***	1.79, 2.29	**1.64** ***	1.49, 1.82
Fall (SON)	**1.12** ***	1.04, 1.22	1.05	0.96, 1.14	**1.20** **	1.06, 1.37	**1.64** ***	1.48, 1.81
Winter (DJF)	**0.81** ***	0.75, 0.88	**0.59** ***	0.53, 0.65	**0.87**	0.76, 1.00	1.03	0.93, 1.15

All results were modeled using the glmmTMB package.

a Negative binomial with type 1 variance.

b Type 2 variance (see Methods for more information).

Significance codes: ***P < 0.001; **P < 0.01; *P < 0.05; “ ” *P* < 0.1.

Exp (Est.) indicates exponentiated estimate.

### Regression analysis results

Multiple regression models were evaluated to determine the optimal approach for assessing associations between geographic coverage of private wells, karst geology distribution, and enteric illness incidence. A zero-inflated negative binomial model with a random intercept for county and type 1 or type 2 variance structure demonstrated the best fit across all pathogens (Table S1 https://links.lww.com/EE/A436). All subsequent analyses utilized this model specification. No statistically significant interaction was detected between the proportion of county area served by private wells and the proportion underlain by karst geology for any pathogen (Table 2).

**Table 2.** Incidence rate ratios from the zero-inflated negative binomial models for the relationship between area served by private wells and area underlain by karst geology and enteric disease (circa 2010-2019) (for each quartile increase in area served by private wells or area underlain by karst, there is an IRR-fold increase in cases of illness)

	*Campylobacter*				*Salmonella*				*Cryptosporidium*				*Giardia*			
*Fixed Effects*																
	M1		M2		M1		M2		M1		M2		M1		M2	
	IRR	95% CI	IRR	95% CI	IRR	95% CI	IRR	95% CI	IRR	95%CI	IRR	95%CI	IRR	95%CI	IRR	95%CI
Quartile Area no PWS	**1.29** ***	1.15, 1.45	**1.35** ***	1.21, 1.51	1.04	0.97, 1.11	1.05	0.99, 1.11	**1.25**	0.99, 1.56	**1.33** *	1.07, 1.66	**1.29** ***	1.15, 1.46	**1.25** **	1.11, 1.42
Quartile Area Karst			**1.21** **	1.08, 1.35			1.04	0.97, 1.11			**1.36** **	1.09, 1.69			**0.87** *	0.77, 0.99
Random effects																
*V*(*b*_1_)	0.299		0.273		0.062		0.060		1.028		0.916		0.308		0.292	

M1 indicates model 1 results (no PWS only); M2, model 2 results (no PWS and area underlain by karst); *V*(*b*_1_), variance of the random intercept for county

*P* values for the multi-level negative binomial model were calculated using glmmTMB and gtsummary packages.

Signif. codes:

*** *P*-trend < 0.001;

** *P*-trend < 0.01;

* *P*-trend < 0.05; “.”*P* < 0.1.

#### Campylobacter

Statistically significant associations were observed between campylobacteriosis case counts and both the proportion of county area not served by public water supply and the proportion underlain by karst geology. Each 25% increase in area lacking public water supply was associated with a 35.2% increase in campylobacteriosis cases (IRR = 1.35 [95% CI: 1.21, 1.51]), while each 25% increase in karst coverage corresponded to a 20.8% increase in cases (IRR = 1.21 [95% CI: 1.08, 1.35]) (Table 2).

#### Salmonella

No statistically significant associations were found between salmonellosis case counts and either the proportion of county area not served by public water supply or the proportion underlain by karst geology.

#### Cryptosporidium

As presented in Table 2, statistically significant associations were identified between cryptosporidiosis case counts and both the proportion of county area lacking public water supply and the extent of karst geology. Each 25% increase in area without public water supply corresponded to a 33% increase in cryptosporidiosis incidence (IRR = 1.33 [95% CI: 1.07, 1.66]), while each 25% increase in karst coverage was associated with a 36% increase in incidence (IRR = 1.36 [95% CI: 1.09, 1.69]).

#### Giardia

A positive association was observed between giardiasis case counts and the proportion of county area not served by public water supply, while a negative association was found with karst geology coverage. Each 25% increase in area lacking public water supply was associated with a 25% increase in giardiasis cases (IRR = 1.25 [95% CI: 1.11, 1.42]), whereas each 25% increase in karst coverage corresponded to a 13% decrease in cases (IRR = 0.87 [95% CI: 0.77, 0.99]) (Table 2).

## Discussion

We found that PA incidence rates (per 100,000) for 2010–2019 were nearly twice the US rates for infections due to *Cryptosporidium* (4.9 vs. 2.5) and 1.5 times higher for *Campylobacter* (31 vs. 20), but lower for *Salmonella* (9.2 vs. 15.2) and comparable for *Giardia* (5.9 vs. 6.0).^32^ Overall, statistically significant spatial clusters across PA explained the highest proportion of *Cryptosporidium* (30.6%) and *Campylobacter* (17.8%) cases. Importantly, we found that rates of illness due to *Campylobacter*, *Cryptosporidium*, and *Giardia* were positively and significantly associated with the prevalence of private well ownership. In addition, karst geology, a geology more prone to facilitate well water contamination, was also positively associated with an increased incidence of illnesses due to *Campylobacter* and *Cryptosporidium*.

### Time–space relationships of enteric disease

We found statistically significant spatial clusters for all pathogens across the state of PA, with some pathogen clusters overlapping one another. When visualizing the IRRs at the county level, Sullivan County had an IRR >2.0 for all four pathogen, and Warren and Lycoming counties had an IRR > 2.0 for all pathogens but *Cryptosporidium*.

#### Cryptosporidium and Giardia

In our temporal analysis, most cases of giardiasis and cryptosporidiosis occurred in summer and fall. Out of the five outbreaks identified due to *Giardia,* three occurred in the winter and two in the spring. For the two *Cryptosporidium* outbreaks, one occurred in spring and one in winter. Lal et al.^33^ utilized a Bayesian spatiotemporal framework to identify outbreaks due to *Cryptosporidium* and *Giardia* in New Zealand. They found no seasonal pattern in outbreak probability for giardiasis, while for cryptosporidiosis, they observed outbreaks in the spring, which aligns with our findings. Our results for *Giardia* are similar to a study in Ontario, Canada, that found that giardiasis rates peaked in late summer to early fall.^34^

After travel and immunocompromising conditions, risk factors for *Cryptosporidium* include waterborne transmission (such as contact with recreational water, wastewater, and consumption of untreated water), contact with animals, and food exposures (meat consumption, raw milk, and dishes consumed outside the home).^35^ For giardiasis, primary risk factors for *Giardia* outbreaks in high-income countries are contaminated drinking water and contact with young children wearing diapers.^36^ These risk factors have spatial and temporal features that could affect exposure patterns, which may explain our findings, such as increased exposure to recreational water in summer months or increased drinking water contamination due to snowmelt and rainfall events in spring.

#### Campylobacter and Salmonella

Documented risk factors for exposure to *Campylobacter* include recreation, untreated drinking water/ well water exposure, consumption of raw milk, undercooked chicken, and visiting/ living on a farm. While risk factors associated with *Salmonella* infection in the general population include travel, food (eggs, composite foods, and meat), and untreated water.^37^ These exposures can be linked both temporally (i.e., water recreation and undercooked chicken in summer) and spatially (i.e., raw milk consumption is more common in certain areas) with infections.

We observed six outbreaks of *Campylobacter* and 12 outbreaks of *Salmonella* in our analyses. Cases in the summer months were also very high for both illnesses. In PA, many *Campylobacter and Salmonella* outbreaks have been linked to raw milk consumption, which can be clustered in space and time.^38^ We also observed spatial trends where Warren, Sullivan, and Lycoming counties had the highest IRRs of both diseases (Figures 3 and 4). Additionally, the counties that border New York State in the north of PA all had elevated IRRs of both enteric infections. These counties all have lower median income compared to the rest of the State and lower levels of education; they also have reported drinking water violations.^39^

In New Zealand, campylobacteriosis rates were found to be heterogeneous both spatially and temporally, and they found a strong seasonal component to the illnesses.^40^ *Campylobacter* and *Salmonella* were found to have peak incidence rates in summer in a study in New Brunswick, Canada, which was similar to our findings.^41^

### Enteric disease and private wells

#### Epidemiological studies

We found an association between private well usership and rates of illness due to *Campylobacter*, *Cryptosporidium*, *and Giardia* in PA. A recent study analyzing a large water quality dataset from Ontario, Canada, had similar results, where they suggest that *Campylobacter* and *Cryptosporidium* cases might be groundwater-related.^42^ Murphy et al.^4^ estimated that 1.1% of cases of *Giardia*, 4.3% of cases of *Campylobacter*, and 45% of cases of *Cryptosporidium* in Canada were attributed to private wells. Fullerton et al.^43^ found that consumption of private well water was associated with *Campylobacter* infections in infants 0–6 months admitted to hospitals in the United States. Globally, 31 published groundwater outbreaks have been attributable to *Campylobacter*.^8^

Although, in our analysis, there was no association found between private wells and incidence rates of salmonellosis, in a case–control study of children under the age of 5 in the state of Washington, the odds of children who had confirmed illnesses due to *Salmonella* were more likely to be exposed to a private well as their home water source (OR = 6.5 [95% CI: 1.4, 29.7]).^44^ In a study assessing the possible association between the prevalence of private wells and the incidence of salmonellosis over a decade at the zip code level in Maryland, United States, in 2020, a significant positive association was found (RR = 1.35 [95% CI: 1.11, 1.63]). If we had assessed private well occurrence at the ZCTA level, it is possible we may have also seen this relationship.

A UK-based study of cryptosporidiosis and giardiasis suggested that private well usage may contribute to giardiasis transmission, as evidenced by the increased odds of cases being classified as rural. The authors identified small community water sources as a risk factor for both infections.^45^ In a review by Murphy et al.,^8^ they noted 7 and 16 groundwater outbreaks globally have been associated with *Giardia* and *Cryptosporidium*, respectively.

#### Detection of Pathogens in Private Wells

All four pathogens examined in this analysis have been detected in private well water. In Wisconsin, Stokdyk et al.^9^ detected all four of our pathogens of interest in well water.^9^ Human pathogens were identified in 48% of sampled wells (n = 138), with *Cryptosporidium* being the most prevalent pathogen, followed by *Salmonella*. In a separate study, Stokdyk et al.^10^ detected *Cryptosporidium* in 40% of 145 wells sampled in Minnesota. In a review of North American groundwater studies, the majority (60%) of studies that collected samples for pathogen testing found bacterial and protozoan pathogens.^8^

### Geology and well water contamination and disease

Geologic substrates that are highly transmissive, such as fractured granite, gravel, or karst, can allow for rapid transport of pathogens to groundwater.^46^ Although there are many papers describing the occurrence of pathogens within karst aquifers, to our knowledge, there is little research that has examined geographic locations of wells underlain by karst formations and associations with spatial clustering of enteric disease.

Gastrointestinal illness or well water contamination attributable to karst geology has been documented for multiple pathogens. In eastern Tennessee, proximity to karst terrain demonstrated an inverse relationship with *E. coli* O157:H7 and cryptosporidiosis infections.^47^ In Ireland, an assessment of monitoring wells from 2011 to 2020 revealed that karst limestone geology was associated with generic *E. coli* contamination (OR = 2.76, *P* = 0.03).^48^ In Ohio, which borders Pennsylvania to the west, a positive association between campylobacteriosis and karst geology was identified.^49^

The findings of the current study, demonstrating increased *Campylobacter* and *Cryptosporidium* cases in areas underlain by karst, align with existing literature suggesting that well water from karst aquifers can serve as a source of enteric disease transmission. However, giardiasis cases exhibited an inverse association with karst geology, potentially indicating that *Giardia* infections in these regions may be influenced by other spatially correlated factors (e.g., reduced density of animal or human reservoirs). The spatial distribution of *Giardia* clusters and karst areas demonstrates less overlap compared with the concordance observed between karst and *Campylobacter* and *Cryptosporidium* clusters (Figures 1 and 3).

### Strengths and limitations

Strengths of this work include that PA was an optimal state to conduct this analysis due to the large number of households that use private wells,^50^ the varied land use (peri-urban, rural, urban), and the presence of karst aquifers over an estimated 20% of the state.^26^ Another strength is the use of spatial modeling methods, which builds on the previously examined relationship between *Campylobacter* and private drinking water in Maryland,^12^ while adding geology, a factor that has been identified as leading to outbreaks associated with untreated groundwater in the United States.^3^

A primary limitation of this study was that the available data could not identify the true source of exposure for individual cases. We used the location of the home address reported by the individual at the time they were diagnosed. This means that cases could be exposed to illness through water, food, or other exposure routes at another location outside the home-assigned ZCTA (e.g., at work, school, daycare, restaurant, campground, etc.). As a result, these cases will be misclassified using the home address method used in our analyses. The mode of transmission of the cases (e.g., foodborne or waterborne) was not accounted for in this analysis, nor was whether the case was linked to a known outbreak, as this data was not available. However, this information may not have substantially influenced the results for *Cryptosporidium*, *Campylobacter*, and *Giardia*, as over 60% of outbreaks attributed to these pathogens in Pennsylvania are classified as either waterborne or of unknown source according to the National Outbreak Reporting System (Table S6 https://links.lww.com/EE/A436 and National Outbreak Reporting System Dashboard | CDC, date last accessed May 28, 2024).^32^ Conversely, in the case of illnesses due to *Salmonella*, which have a higher fraction attributed to foodborne outbreaks, the lack of information on the transmission source could have greatly improved our risk estimates related to water, because we could have excluded those cases from the analysis.

The health data used in this study have been collected through passive surveillance. The widely noted limitations of passive surveillance are underreporting, underdiagnosis, lack of representativeness of reported cases, and lack of timeliness.^51^ A limitation of our analysis is that many cases of interest that would have been included in this analysis often go unreported.^1^

Lastly, we relied on the public water service boundary information and assumed that those not served by a PWS relied on private wells. Our approach assumed that an area “not in the PWS area” was relying on private wells and that the same proportion of area served by private wells is the same as that of families in a census tract. Although this assumption is an imperfect proxy measure, it is the best data we had available, as there are currently no complete registries of drinking water sources in PA for those using private wells. Finally, we had no information on individual wells—neither their construction, type, nor current state of repair/disrepair. Taken together, the data limitations and the associations we observed suggest the need to capture improved information on cases regarding private well use as a potential source of sporadic cases of enteric disease.

## Conclusions

We presented a novel spatiotemporal analysis of enteric disease. Our work contributes to a growing body of epidemiological evidence that consumption of private well water contributes to a substantive proportion of enteric disease burden. Our findings are relevant not only to approximately 25% of the population of the State of Pennsylvania but more broadly to countries where a large proportion of the population is relying on private, unregulated well water supplies. Specifically, the findings in this analysis suggest:

1. That federally unregulated private wells may be contributing to enteric diseases due to *Campylobacter*, *Cryptosporidium*, and *Giardia* in PA.
2. The occurrence of karst geology within a county may be contributing to the burden of campylobacteriosis and cryptosporidiosis.
3. That the central portion of the state, especially during the summer months, is experiencing a high burden of enteric disease for all four pathogens.

Future work should include a spatiotemporal assessment that allows for expansion of the model to include other demographic information (e.g., income, race, education) and environmental risk factors for each of these pathogens (e.g., land use, agricultural activities), and to determine if other water exposures, such as recreation, may be driving some of these summer illnesses.

Lastly, our work provides evidence to support interventions to improve private well water management as well as enteric disease reporting. We have the following recommendations:

1. Enteric disease reporting should include additional variables (e.g., private well exposure, date of symptom onset).^52^
2. Public health interventions should be targeted at the management and treatment of private well water in spatial areas of high risk (areas with increased disease, karst geology)
3. Interventions should also be targeted at health practitioners (pediatricians, primary care physicians) so that they understand the increased risks of patients served by private well water, particularly those in areas with vulnerable geology.

## Conflict of interest statement

H.M.M. reports financial support was provided by the Pennsylvania Department of Health. H.M.M. reports a relationship with Steve Harvey Law that includes paid expert testimony. H.M.M. has also received funding from the National Institutes of Health Institute of Allergy and Infectious Diseases (R01AI153376) to study the impact of drinking untreated well water on enteric disease in Pennsylvania. The other authors declare that they have no known competing financial interests or personal relationships that could have appeared to influence the work reported in this paper.

## ACKNOWLEDGMENTS

We would like to acknowledge the support of the Pennsylvania Department of Health’s Division of Surveillance for the timely sharing of a clean dataset of the enteric disease data from 2010 to 2019 on which this manuscript is based. We would also like to thank Dr. Jerry Fagliano for his review of the manuscript and Dr. Regai Yucel for his assistance with some of the statistical analyses. We would also like to acknowledge that some sections of this manuscript were proofread using Claude.ai for grammar and clarity improvement.

## Author contributions

M.W. contributed to conceptualization, data curation, formal analysis, investigation, methodology, visualization, as well as drafting the original manuscript and reviewing and editing the final manuscript. K.H. and E.C. contributed to methodology, supervision, visualization, and editing and reviewing the final manuscript. R.T.W. contributed to supervision, visualization, and reviewing and editing the final manuscript. H.M.M contributed to conceptualization, funding acquisition, methodology, resources, supervision, validation, visualization and drafting the manuscript, and reviewing and editing the final manuscript.

## Ethics approval

We performed a secondary analysis of illness case data provided by the PA Department of Health. This research was approved by the PA Department of Health’s and Temple University’s Institutional Review Boards (Temple IRB Protocol # 28345).

## Data availability

The authors do not have permission to share the illness data provided by the State of Pennsylvania used in the analyses in this paper. The data used to generate the public water supply boundaries can be found below. The details of all the R packages used and data analysis methods are described in the manuscript and supplemental materials. These data are supplied by the Pennsylvania Department of Environmental Protection. Public water supplier’s service area data from the year 2018 were accessed and used via the Pennsylvania Spatial Data Access and may be accessed here: Public Water Systems—Public Water Supplier Service Areas | PA Department of Environmental Protection (arcgis.com)—https://newdata-padep-1.opendata.arcgis.com/datasets/PADEP-1::public-water-systems-public-water-supplier-service-areas/about

## Supplementary Material


