# Supplementary material for: Are cases of enteric disease due to Salmonella, Campylobacter, Giardia, and Cryptosporidium associated with well water?
Source: Environ Epidemiol. 2026 Jun 16;10(4):e499. doi: 10.1097/EE9.0000000000000499 (PMC13275149; doi:10.1097/EE9.0000000000000499)
Supplement: Supplementary file 1 [file ee9-10-e499-s001.pdf]

Supplementary Materials

Are cases of enteric disease due to *Salmonella, Campylobacter*, *Giardia*, and *Cryptosporidium* associated with drinking water source?

Miriam Wamsley[1], Kevin Henry[2], Robin Taylor Wilson[1] , Eric Coker[3], and Heather M. Murphy [1,4*]

1 Department of Epidemiology and Biostatistics, Temple University Philadelphia, PA, USA
2 Department of Geography, Environment, and Urban Studies, Temple University Philadelphia, PA, USA
3 British Columbia Centre for Disease Control, Vancouver, BC, Canada
4 Water, Health and Applied Microbiology Lab, Department of Pathobiology, University of Guelph, Guelph, ON, Canada

*****Correspondence:
Heather M. Murphy
Department of Pathobiology, Ontario Veterinary College,
University of Guelph
50 Stone Rd. East, Guelph, ON Canada
heather.murphy@uoguelph.ca

Text S1.- Spatial Analysis

The software evaluated the number of reported cases within each ZCTA relative to surrounding ZCTAs at progressively increasing radial distances from the ZCTA centroid (Kulldorff, 2018; Kulldorff et al., 2005). This iterative process identified relative risk values exceeding those attributable to random variation, with the maximum scanning window encompassing up to 5% of the state population (excluding Philadelphia). Geographic data were matched to illness counts using ZCTA codes. We employed a discrete Poisson model to identify high-risk clusters, defined as geographic areas with elevated relative risk of illness by ZCTA, and adjusted for the population within each ZCTA.

In this Poisson-based SaTScan analysis, a cluster was identified when the observed incidence significantly exceeded the expected incidence, calculated using the 2010–2019 statewide mean incidence rate for each pathogen. Statistical significance was assessed through Monte Carlo simulations, where cases were randomly redistributed across the study area. The null hypothesis assumed no spatial variation in incidence rates, with cases randomly distributed throughout the state (Kulldorff, 2018; Kulldorff et al., 2005). Clusters were reported if they exhibited a Gini coefficient of 1.0 and a p-value ≤ 0.1. The Gini coefficient, derived from the Lorenz curve and implemented within SaTScan to determine cluster reporting thresholds, ranges from 0 to 1 (Gastwirth, 1972; Han et al., 2016). A coefficient of 0 indicates no clustering (uniform distribution of illness rates across geographies), while a coefficient of 1 indicates maximal clustering (heterogeneous distribution of illness rates across geographies) (Gastwirth, 1972; Han et al., 2016).

Text S2- Temporal Analysis

We evaluated a 10-year dataset for each pathogen to assess temporal patterns and seasonality. First, to examine trends over time and determine whether seasonality warranted inclusion as a covariate in our models, we decomposed weekly time series data for each pathogen into constituent components using classical decomposition methods implemented in the feasts package in R (Supplementary Figures S1–S4) (O'Hara-Wild et al., 2022). Second, to characterize the association between seasonal variation and illness counts for each pathogen, we employed a zero-inflated negative binomial regression model. The total state population, excluding Philadelphia, served as the denominator for each year and was incorporated as an

offset term in the model (Equation 1), denoted as log Pop_t, where t = 1, 2, …, 523 represents weekly intervals across the decade. Season was modeled using four dummy variables with spring as the reference category. The seasonal classifications were defined as follows: winter (December–February), spring (March–May), summer (June–August), and fall (September–November).

Equation 1. $\log\ (Count_t) = \ (\beta_0) + \beta_1 season_t + \log(Pop._t\,)$

Third, to estimate the occurrence and frequency of outbreaks, we defined an outbreak as a week in which observed case counts exceeded model predictions. We fit a negative binomial regression model using the Model Temporal Trending package (version 0.0.3) with ten years of weekly count data for each pathogen (Schumacher et al., 2021). The model incorporated a Fourier matrix term for seasonality from the forecast package (representing sinusoidal waves decomposed into sine and cosine values for each of 52 weeks) and a linear time term to account for decadal trends (Hyndman et al., 2023). We plotted weekly estimates with corresponding confidence intervals and prediction intervals from the negative binomial model outputs. An outbreak was defined as a week in which illness counts exceeded the upper bound of the 95% prediction interval. Prediction intervals, which accommodate greater uncertainty than confidence intervals, ensure that identified outbreaks are unlikely to result from random variation (Taylor & Bunn, 1999).

Text S3- Regression Analysis

We employed an ecological study design utilizing multiple zero-inflated negative binomial regression models with random intercepts for county to assess the effect of the percentage of county area underlain by karst geology, and the percentage of geographic area lacking access to public water supply (i.e., reliance on private wells) on weekly counts of new illness cases by county for each pathogen. When both the percentage of area served by private wells and the percentage of area underlain by karst were statistically significant, we assessed their interaction effect on county disease counts. These models were fitted separately for each pathogen. The model is described in Equation 2:

$Equation$ 2.

$$\log\ (Count_{it}) = \ (\beta_0 + b_i) + \ \beta_1 area\ wells_i + \beta_2 area\ karst_i + (\beta_1 area\ wells_{it} \times \beta_2 area\ karst_{it}) + \log(Pop_{\cdot it}\ )$$

where *i = 1, 2,…, 66*, denoting county, and *t = 1, 2,…, 523* indicates week over a decade

County-specific annual population estimates served as offset terms in the model, denoted as log Pop_{it}. The county-specific random intercept, denoted as b_i, was included to partially account for unmeasured risk factors that vary geographically, including geophysical characteristics and heterogeneity in healthcare access and disease reporting practices across the state (Gelman et al., 1999) Fixed effects included: quartiles of area served by private wells (no public water supply), denoted as area wells; quartiles of county area underlain by karst, denoted as area karst; and an interaction term between these two variables.

To accommodate the numerous weeks in which counties reported zero cases, we employed a zero-inflated modeling framework. Zero-inflated models are two-component models: one component models the probability of observing zero cases, while the second component assesses the relationship between non-zero counts and covariates (in this case, area served by private wells and area with karst geology). The zero-inflated negative binomial model is summarized in Equation 3, where y represents the number of cases per county per week and E(y = k) represents the expected value:

Equation 3.

$E(y = k|cases\ reported)$:

$$E(count = k) = P\ (\text{zero cases reported }) * 0 + P\ (cases\ reported) * E(y = k|cases\ reported)$$

We utilized the glmmTMB package in R to fit and compare various zero-inflated models for each pathogen, including Poisson, negative binomial with type 1 variance (NB1), and negative binomial with type 2 variance (NB2) specification (Brooks et al., 2022) in NB2 models, variance increases quadratically with the mean, whereas in NB1 models, variance increases linearly with the mean (Brooks et al., 2017; Hardin & Hilbe, 2018). Model fit was evaluated using the Akaike information criterion (AIC), with the model exhibiting the lowest AIC selected as the best-fitting model (Supplementary Table S1). We

calculated incidence rate ratios (IRRs) and their corresponding 95% confidence intervals. All analyses and visualizations were performed in R version 4.2.2 (R Core Team, 2022).

Text S4- Results Spatial Clusters

3.1.1 *Campylobacter*

SaTScan analysis identified nine statistically significant spatial clusters exhibiting higher-than-expected campylobacteriosis incidence relative to the statewide average (Supplementary Figure S1). All clusters demonstrated statistical significance ($p < 0.001$), with relative risks ranging from 1.62 (cluster 9) to 3.31 (cluster 2) (Supplementary Table S3). Three clusters with radii exceeding 50 km were concentrated in the northern region of the state. These clusters correspond to areas of elevated incidence rates depicted in Supplementary Figures S1–S2 and Figure 3.

3.1.2 *Salmonella*

Six spatial clusters of elevated salmonellosis incidence were identified (Supplementary Figure S1). One particularly large cluster, with a radius exceeding 100 km, was located in the north-central region (Supplementary Table S3). Kriged surfaces revealed smaller-scale spatial variability throughout the state (Supplementary Figure S2), with Figure 4 demonstrating lower county-level incidence rates in the southeastern region.

3.1.2 *Cryptosporidium*

SaTScan analysis identified ten statistically significant clusters of cryptosporidiosis ($p < 0.1$) (Supplementary Figure S1). These clusters were concentrated in the western and central portions of the state, including rural areas surrounding Pittsburgh and regions north and west of Harrisburg (Supplementary Table S3). The spatial distribution of smoothed ZCTA-level incidence rates (Supplementary Figure S2) corresponded closely with SaTScan cluster locations and county-level incidence rate ratios (Figure 5), particularly elevated rates in the south-central region (corresponding to clusters 2 and 6) and the northeastern quadrant (corresponding to cluster 4).

3.1.3 *Giardia*

Thirteen statistically significant spatial clusters of giardiasis were identified ($p < 0.05$) (Supplementary Figure S1, Supplementary Table S3). Relative risks ranged from 1.86 (cluster 15) to 10.35 (cluster 17). The highest burden of giardiasis in Pennsylvania was concentrated in the northwestern quadrant (Figure 6 and Supplementary Figure S2).

S1 Table- Model selection results for best fit using the Akaike information criterion (AIC), whereby the lowest AIC models were determined to have the best fit.

	Salmonella		Salmonella with karst		Campylobacter		Campy with karst		Giardia		Giardia with karst		Cryptosporidium		Crypto with karst	
Fit	AIC	df	AIC	df	AIC	df	AIC	df	AIC	df	AIC	df	AIC	df	AIC	df
Zero inflated negative bionomial (Type 2)	0	5	0	6	0	5	0	6	7.8	5	7.8	6	21.6	5	21	6
Zero inflated negative bionomial (Type 1)	46.9	5	47.1	6	8.1	5	8.4	6	0	5	0	6	0	5	0	6
Zero inflated Poisson	219.6	4	219.4	5	213.1	4	200.9	5	21.4	1	16	5	39.7	4	39.1	5

S2 Table. Table of proportion of area not served by public water supply by census tract

NAME	FIPS	POPULATION	HOUSEHOLDS	SQMI	Proportion of county Area with no PWS
Allegheny County	42003	1,221,810	533,960	744.26	0.0933
Delaware County	42045	568,500	208,700	185.06	0.1155
Montgomery County	42091	843,278	307,750	487.21	0.2445
Westmoreland County	42129	355,134	153,650	1035.93	0.3704
Fayette County	42051	131,508	55,997	798.73	0.4225
Washington County	42125	211,984	85,089	860.85	0.4739
Bucks County	42017	639,420	234,849	620.35	0.5464
Chester County	42029	532,096	182,900	759.2	0.5569
Greene County	42059	37,243	14,724	577.98	0.5745
York County	42133	455,952	168,372	910.74	0.5984
Beaver County	42007	165,968	71,383	444.03	0.6297
Northampton County	42095	312,074	113,565	377.15	0.6483
Lehigh County	42077	372,233	133,983	348.22	0.6871
Clearfield County	42033	80,266	32,288	1154.57	0.7506
Lackawanna County	42069	215,973	87,226	465.16	0.7764
Indiana County	42063	86,101	35,005	834.39	0.7935
Cambria County	42021	133,913	58,950	692.73	0.7965
Lawrence County	42073	87,945	37,126	361.78	0.8069
Cumberland County	42041	255,665	93,943	550.31	0.8086
Luzerne County	42079	322,054	131,932	905.92	0.8144
Dauphin County	42043	280,234	110,435	558.16	0.8162
Lebanon County	42075	145,257	52,258	362.5	0.8428
Lancaster County	42071	552,587	193,602	983.84	0.8451
Armstrong County	42005	66,401	28,713	663.97	0.8456
Northumberland County	42097	91,329	39,242	477.23	0.8459
Erie County	42049	273,523	110,413	804	0.8466
Schuylkill County	42107	143,461	60,192	782.76	0.8476
Franklin County	42055	157,402	58,389	772.9	0.8536
Butler County	42019	195,549	72,835	795.66	0.865
Blair County	42013	125,035	52,159	526.55	0.8669
Berks County	42011	426,258	154,356	865.75	0.8694
Centre County	42027	167,509	57,573	1113.02	0.88
Adams County	42001	105,930	38,013	521.69	0.8824
Clarion County	42031	39,659	16,128	609.84	0.9112

Monroe County	42089	175,063	61,091	617.11	0.9137
Mercer County	42085	111,787	46,442	682.56	0.9288
Pike County	42103	59,394	21,925	566.82	0.9304
Union County	42119	44,331	14,765	317.74	0.9309
Somerset County	42111	75,239	31,090	1081.66	0.9324
Columbia County	42037	66,324	26,479	490.39	0.9371
Mifflin County	42087	47,107	18,743	414.72	0.9388
Elk County	42047	30,568	13,693	831.85	0.9416
Carbon County	42025	65,461	26,684	387.44	0.9477
Montour County	42093	18,414	7,393	132.33	0.9533
Clinton County	42035	38,272	15,151	896.93	0.9559
Crawford County	42039	85,744	35,028	1037.6	0.9566
Venango County	42121	52,230	22,621	682.82	0.9618
Jefferson County	42065	44,111	18,561	656.8	0.9619
Snyder County	42109	41,401	14,750	331.55	0.9633
Wayne County	42127	54,278	20,625	750.78	0.9653
Lycoming County	42081	113,801	46,700	1243.95	0.9672
McKean County	42083	41,303	17,183	984.72	0.9703
Huntingdon County	42061	46,403	17,280	889.27	0.9806
Juniata County	42067	25,729	9,476	393.52	0.9831
Warren County	42123	40,180	17,767	898.71	0.9831
Bedford County	42009	49,157	20,233	1016.88	0.9832
Cameron County	42023	4,872	2,273	398.42	0.9841
Tioga County	42117	40,319	16,727	1137	0.9846
Perry County	42099	47,542	17,903	555.54	0.9862
Bradford County	42015	61,300	25,321	1161.06	0.9865
Susquehanna County	42115	43,750	17,798	832.14	0.9876
Wyoming County	42131	27,692	11,237	405.11	0.9884
Potter County	42105	16,112	7,227	1081.47	0.9906
Forest County	42053	7,562	2,511	430.49	0.9913
Fulton County	42057	15,025	6,014	438.06	0.9923
Sullivan County	42113	6,066	2,777	452.5	0.9968

S3 Table. Results of SaTScan spatial analysis identifying significant clusters of enteric illnesses by pathogen between 2010-2019

Pathogen	Cluster	Radius(km)	P-value	Observed	Expected	Rel. Risk	Population	PAF
Campylobacter	2	51	0.00E+00	1,383	445	3.31	314,345	5.30
	3	60	0.00E+00	961	385	2.59	272,182	3.23
	5	22	0.00E+00	830	372	2.3	262,785	2.57
	6	39	0.00E+00	399	144	2.82	101,704	1.41
	7	56	0.00E+00	668	369	1.84	260,894	1.66
	9	34	0.00E+00	538	337	1.62	237,834	1.12
	10	13	0.00E+00	159	65	2.46	45,914	0.51
	11	20	0.00E+00	464	287	1.64	202,492	0.99
	12	14	4.40E-11	320	196	1.64	138,578	0.68
	13	25	1.20E-04	67	30	2.23	21,270	0.20
	14	24	2.40E-02	43	19	2.25	13,523	0.13
Salmonella	1	101	2.10E-14	597	408	1.49	460,822	1.71
	2	19	1.30E-06	387	275	1.42	310,299	0.99
	3	14	7.00E-06	239	156	1.54	176,409	0.73
	4	54	1.40E-03	412	317	1.31	357,648	0.85
	5	0	3.30E-03	61	30	2.06	33,554	0.27
	6	10	1.30E-02	31	11	2.7	12,972	0.17
Cryptosporidium	2	62	0.00E+00	525	122	4.94	416,959	11.24
	3	35	0.00E+00	242	80	3.18	274,211	4.41
	4	40	0.00E+00	114	23	5.04	79,655	2.42
	5	27	0.00E+00	165	59	2.89	202,149	2.86
	6	44	0.00E+00	202	83	2.51	285,543	3.22
	7	31	0.00E+00	100	26	3.92	89,400	1.97
	9	29	1.30E-10	77	27	2.91	92,099	1.34
	10	28	9.10E-10	89	35	2.57	120,586	1.44
	11	21	1.10E-04	41	14	2.87	49,271	0.71
	13	18	2.10E-03	77	40	1.93	138,299	0.98
Giardia	2	22	0.00E+00	161	40	4.09	99,915	2.32
	3	38	0.00E+00	139	35	4.07	86,367	2.00
	5	57	0.00E+00	127	42	3.1	103,147	1.64
	6	27	3.40E-14	114	42	2.75	104,168	1.39
	7	58	2.20E-11	106	42	2.56	103,585	1.23
	9	29	2.40E-10	104	43	2.48	105,046	1.18
	10	31	1.40E-06	85	38	2.28	93,142	0.91
	11	29	1.90E-05	87	42	2.11	102,752	0.87
	12	25	1.90E-05	86	41	2.12	101,082	0.87
	13	25	1.30E-03	84	44	1.91	109,572	0.76
	14	9	4.40E-03	78	42	1.89	102,674	0.70
	15	29	2.50E-02	70	38	1.86	93,620	0.62
	17	10	4.50E-02	7	1	10.35	1,673	0.12

*Includes size of cluster, observed vs. expected cases of illness, relative risks and population attributable fraction (PAF)

S4 Table. Temporal regression results identifying outbreaks by enteric pathogen in Pennsylvania between 2010-2019

Pathogen	Cases	Year	Week	Incidence Rate (cases per week)	Lower CI	Upper CI	Lower PI	Upper PI
Giardia	17	2010	W02	7.92	7.33	8.56	2	15
	15	2011	W07	6.68	6.22	7.18	2	13
	17	2011	W19	8.22	7.68	8.78	3	16
	18	2013	W51	7.89	7.44	8.35	2	15
	21	2018	W33	10.41	9.76	11.10	4	19
	13	2011	W13	4.83	4.38	5.33	0	12
Cryptosporidium	12	2017	W11	3.93	3.58	4.31	0	10
	22	2010	W49	11.25	10.58	11.97	4	21
Salmonella	38	2011	W19	21.15	20.07	22.28	10	35
	52	2012	W25	29.94	28.61	31.33	16	48
	20	2012	W51	10.22	9.69	10.79	4	19
	27	2013	W15	14.93	14.23	15.66	6	26
	56	2014	W31	32.72	31.45	34.03	17	52
	31	2014	W44	16.24	15.51	16.99	7	28
	22	2015	W12	11.65	11.09	12.23	4	21
	24	2015	W50	10.79	10.27	11.35	4	20
	26	2018	W13	12.54	11.84	13.28	5	23
	24	2018	W48	11.57	10.91	12.28	4	21
	24	2019	W47	12.43	11.67	13.25	5	23
	55	2012	W04	18.05	17.15	18.99	8	32

Campylobacter	46	2012	W05	18.14	17.25	19.09	8	32
	40	2013	W49	21.82	20.86	22.82	10	37
	37	2014	W04	19.27	18.42	20.16	8	34
	76	2015	W26	41.27	39.64	42.96	21	67
	44	2017	W48	25.53	24.32	26.80	12	43

S5 Table. Peak weeks of counts of illness Statewide for PA 2010 – 2019

Pathogen	Peak weeks of counts over the 523 weeks (ordered by size of peak, high to low, week start Monday)
Campylobacter	52, 26, 17, 87, 75, 10, 262, 13, 20, 105, 40, 7, 523, 48, 19, 3, 44, 8, 2
Salmonella	52, 26, 17, 75, 522, 87, 58, 44, 8, 65, 9, 40, 35, 4, 10, 7, 5, 11, 21
Cryptosporidium	52, 26, 521, 130, 17, 27, 65, 58, 40, 9, 21, 37, 7, 23, 3, 12, 2, 5, 18
Giardia	52, 26, 87, 130, 37, 65, 33, 6, 12, 4, 40, 24, 261, 13, 522, 8, 174, 2, 75

S6 Table. Outbreak Summary for PA, 2010- 2019 (CDC NORS, 2023)

Pathogen	Number of: Total Outbreaks	Multistate Outbreaks	Illnesses	Deaths	Outbreaks due to Food	Outbreaks due to Water	Unknown Cause Outbreaks	Most commonly implicated food vehicle
Campylobacter	138	2	798	0	27	0	87	18 list food vehicle, over half are due to un-pasteurized milk
Salmonella	285	148	15190	30	118	0	76	82 list food vehicle, 15 were poultry, and 15 were fruit
Cryptosporidium	45	0	216	0	1	4	30	1 listed food vehicle, milk
Giardia	68	0	208	0	1	1	53	1 listed food vehicle, mixed salad.

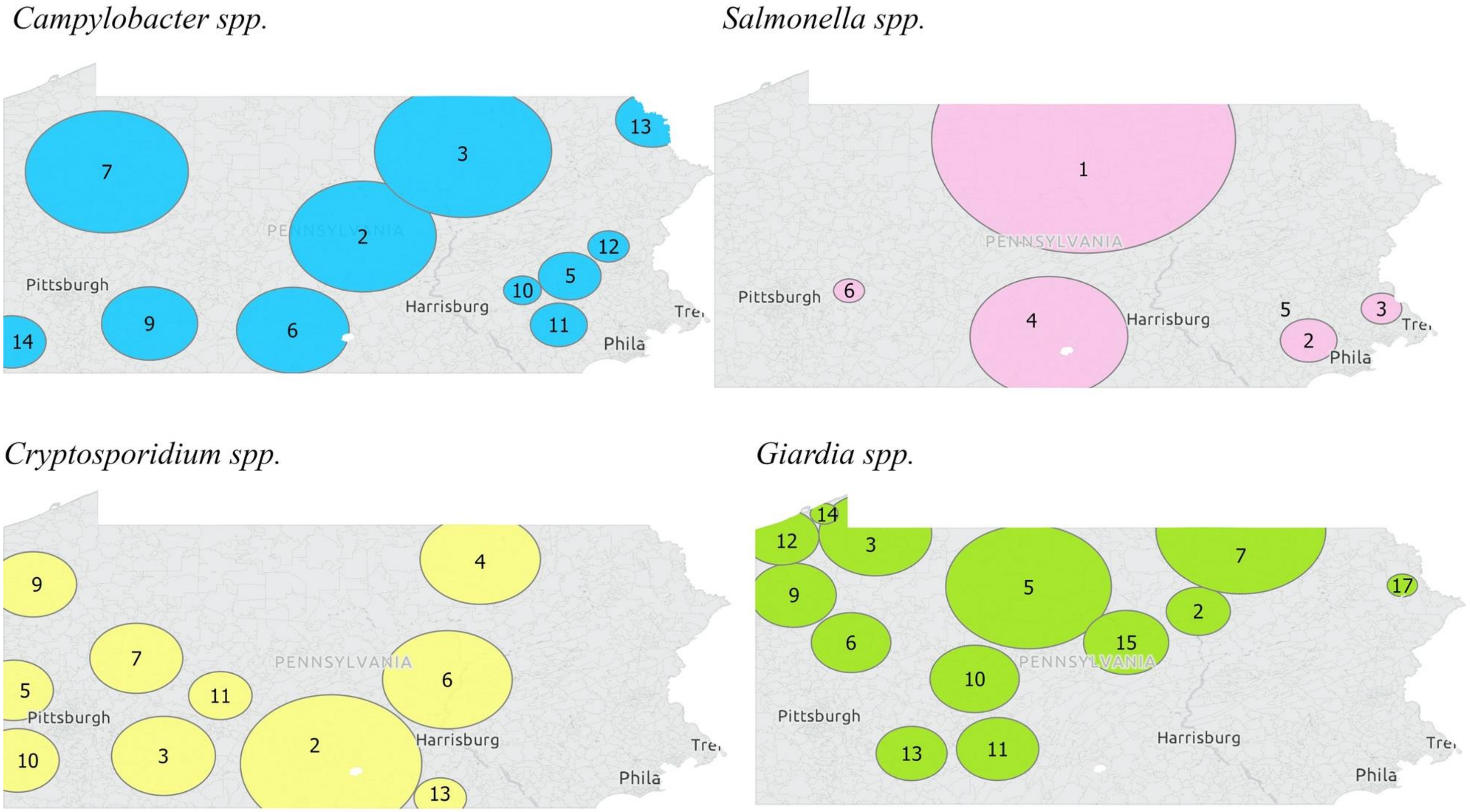

S1 Figure. Spatial clusters of cases *of Campylobacter, Salmonella, Cryptosporidium, Giardia* across PA (circa 2019-2019) (clusters were all significant at p <0.1 and had a Gini Coefficient = 1)

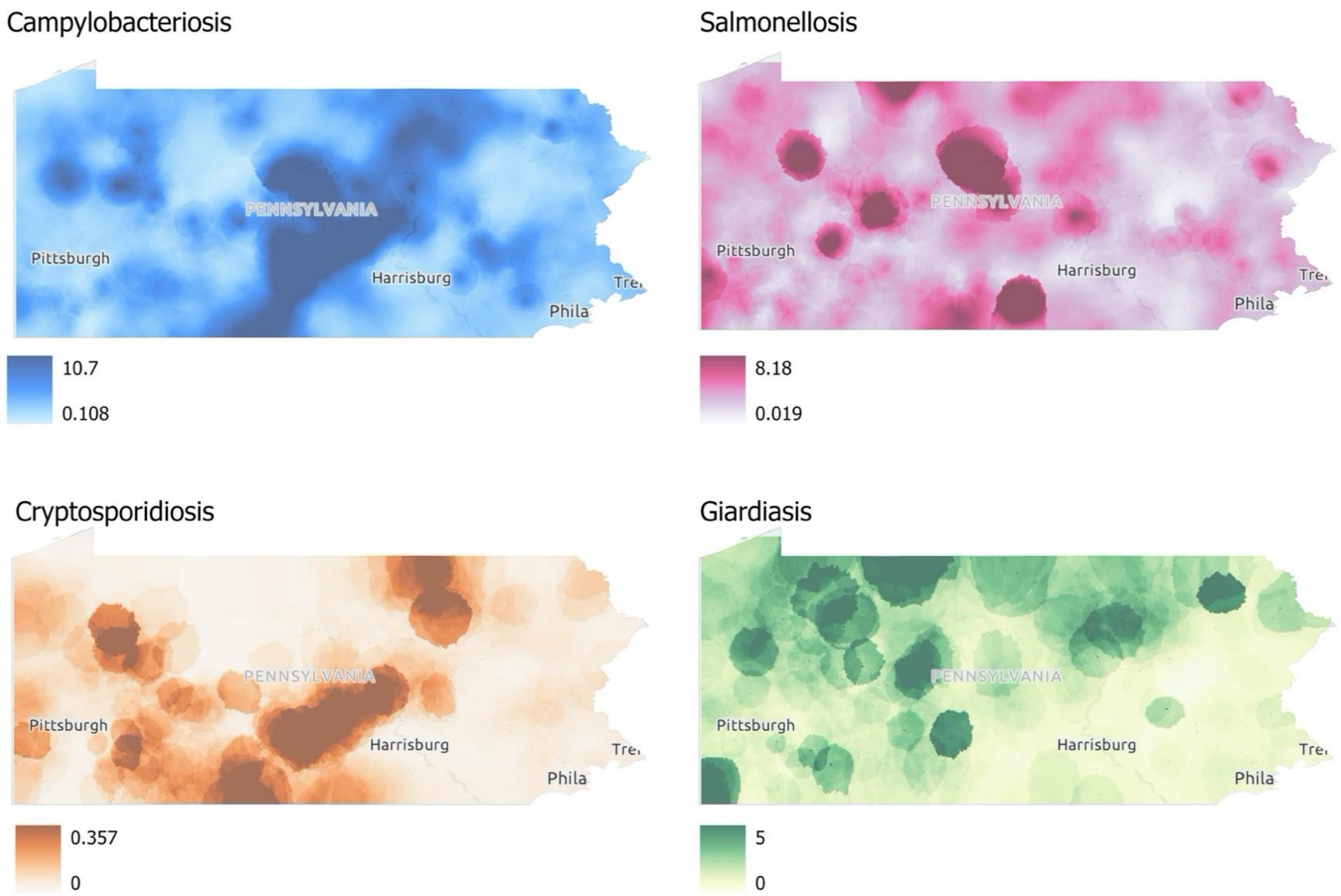

S2 Figure. Average annual incidence rates of reported cases of target illnesses in PA
(circa 2010 -2019)(values represent smoothed data from ZCTA centroids using Kriging) (cases are reported as per 10,000 persons per year) (results excluding Philadelphia)

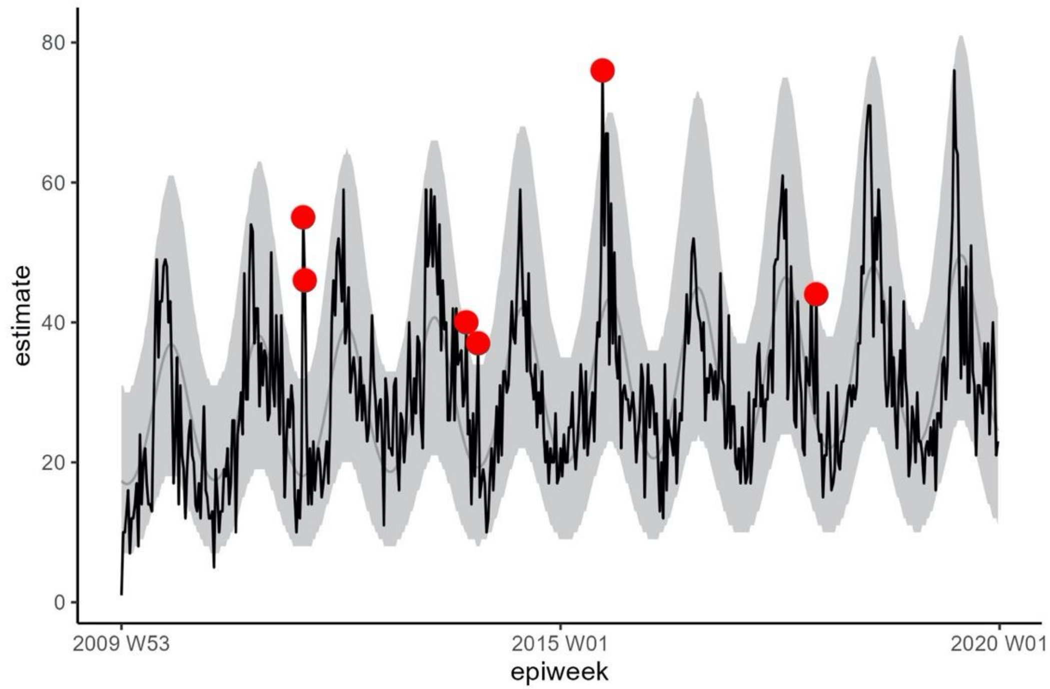

S3 Figure. Temporal regression of weekly reported cases of *Campylobacter* in Pennsylvania between 2010-2019
(black line represents reported cases, grey shading represents 95% prediction interval, red dots indicate outbreaks identified by the model)

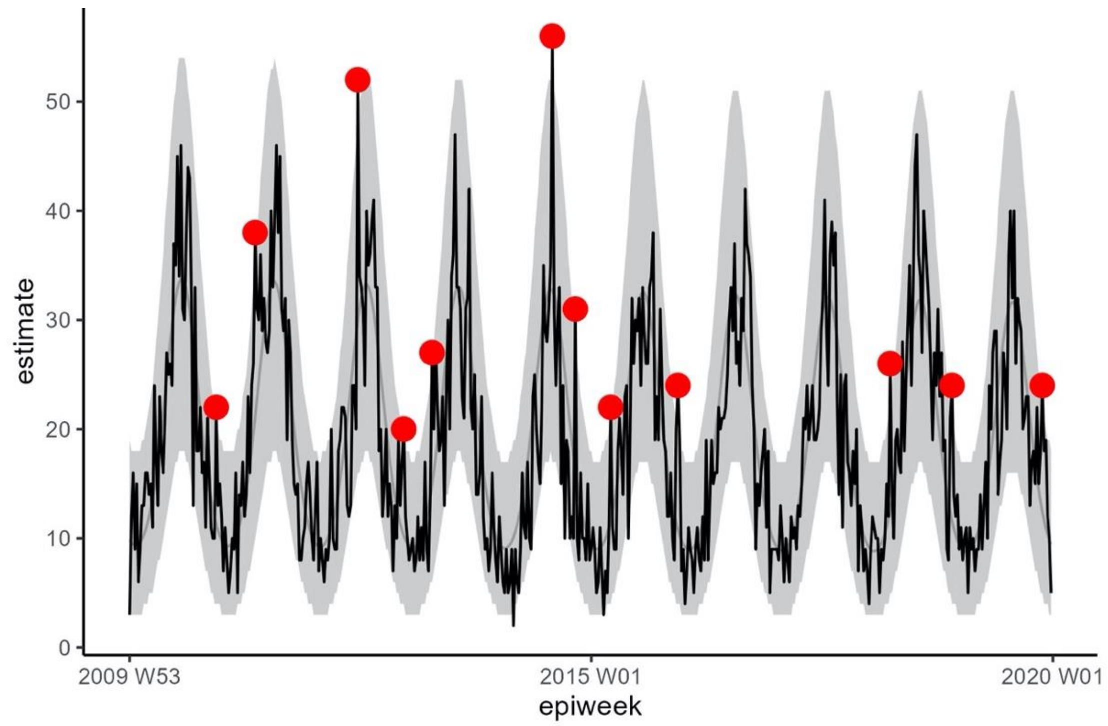

S4 Figure. Temporal regression of weekly reported cases of *Salmonella* in Pennsylvania between 2010-2019 (black line represents reported cases, grey shading represents 95% prediction interval, red dots indicate outbreaks identified by the model)

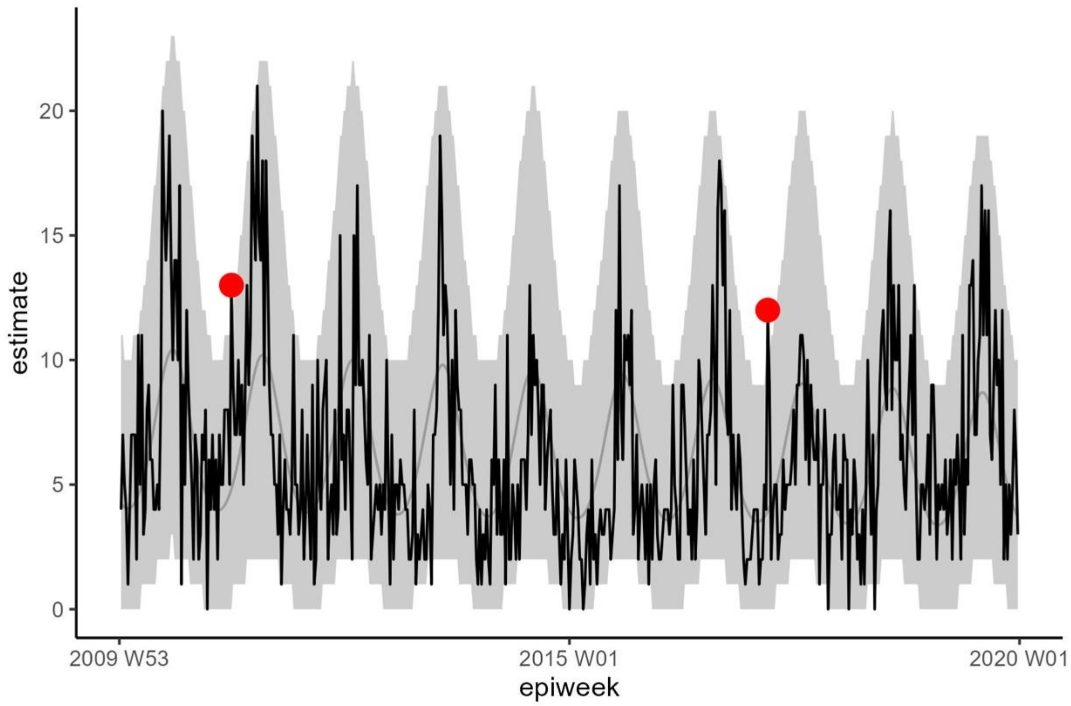

S5 Figure. Temporal regression of weekly reported cases of *Cryptosporidium* in Pennsylvania between 2010-2019
(black line represents reported cases, grey shading represents 95% prediction interval, red dots indicate outbreaks identified by the model)

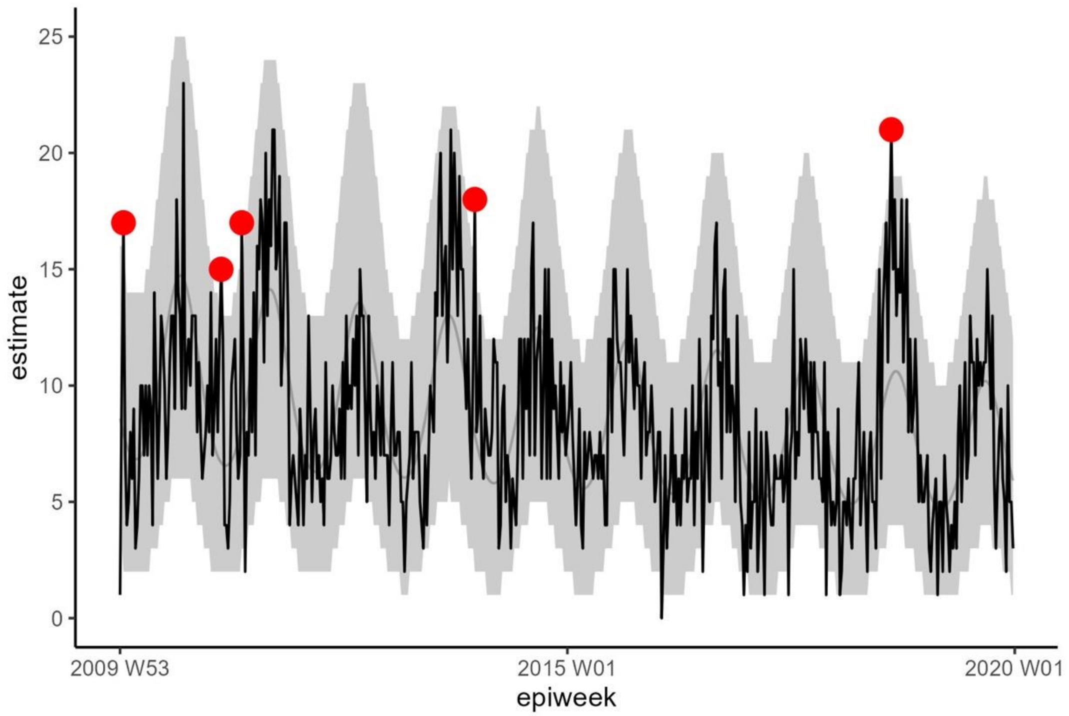

S6 Figure. Temporal regression of weekly reported cases of *Giardia* in Pennsylvania between 2010-2019 (black line represents reported cases, grey shading represents 95% prediction interval, red dots indicate outbreaks identified by the model)
